# Strategies to induce natural killer cell tolerance in xenotransplantation

**DOI:** 10.3389/fimmu.2022.941880

**Published:** 2022-08-22

**Authors:** Kevin J. Lopez, Arthur A. Cross-Najafi, Kristine Farag, Benjamin Obando, Deepthi Thadasina, Abdulkadir Isidan, Yujin Park, Wenjun Zhang, Burcin Ekser, Ping Li

**Affiliations:** Division of Transplant Surgery, Department of Surgery, Indiana University School of Medicine, Indianapolis, IN, United States

**Keywords:** NK cells, NK cell tolerance, xenotransplant, xenotranplantation, tolerance

## Abstract

Eliminating major xenoantigens in pig cells has drastically reduced human antibody-mediated hyperacute xenograft rejection (HXR). Despite these advancements, acute xenograft rejection (AXR) remains one of the major obstacles to clinical xenotransplantation, mediated by innate immune cells, including macrophages, neutrophils, and natural killer (NK) cells. NK cells play an ‘effector’ role by releasing cytotoxicity granules against xenogeneic cells and an ‘affecter’ role on other immune cells through cytokine secretion. We highlight the key receptor-ligand interactions that determine the NK cell response to target cells, focusing on the regulation of NK cell activating receptor (NKG2D, DNAM1) and inhibitory receptor (KIR2DL1-4, NKG2A, and LIR-1) signaling pathways. Inhibition of NK cell activity may protect xenografts from cytotoxicity. Recent successful approaches to reducing NK cell-mediated HXR and AXR are reviewed, including genetic modifications of porcine xenografts aimed at improving pig-to-human compatibility. Future directions to promote xenograft acceptance are discussed, including NK cell tolerance in pregnancy and NK cell evasion in viral infection.

## Introduction

The persistent lack of transplantable organs has resulted in the death of thousands of patients every year. There have been several proposed solutions to address this issue, including manufacturing bioartificial organs (1), 3D printing human organs (2), and transplanting organs from different species into humans, a practice known as xenotransplantation. Xenotransplantation represents one of the most promising approaches. Elimination of major xenoantigens on xenografts by gene-editing tools has proven to be an effective approach to preventing hyperacute xenograft rejection (HXR) (3–6). Earlier this year, the first pig-to-human heart transplantation was performed and supported the patient’s life for two months. In this xenotransplant, HXR was successfully prevented with 10-gene modification, particularly with three major xenoantigens (αGal, Neu5Gc, and Sda) removal in the xenograft (7). Despite this exciting success, xenotransplantation must overcome other barriers before becoming a widespread clinically viable solution. As a result of current advances, the field has shifted towards addressing the next major immunologic barrier: acute and chronic xenograft rejection.

Natural killer (NK) cells are a subset of lymphocytes that not only constitute the innate immune system’s first line of defense but also play a significant role in regulating adaptive immunity (8, 9). NK cells can destroy target cells either directly or *via* antibody-dependent cellular cytotoxicity (ADCC) in the absence of antigen priming (10). NK cell-mediated cytotoxicity may initiate robust adaptive immune responses *via* CD8^+^ T cell priming, antigen-specific CD4^+^ T cell response, and humoral responses (11). NK cells also secrete cytokines and chemokines, which regulate dendritic cells, macrophages, and neutrophils, as well as antigen-specific T cell and B cell function (9, 12, 13).

**Figure 1:**
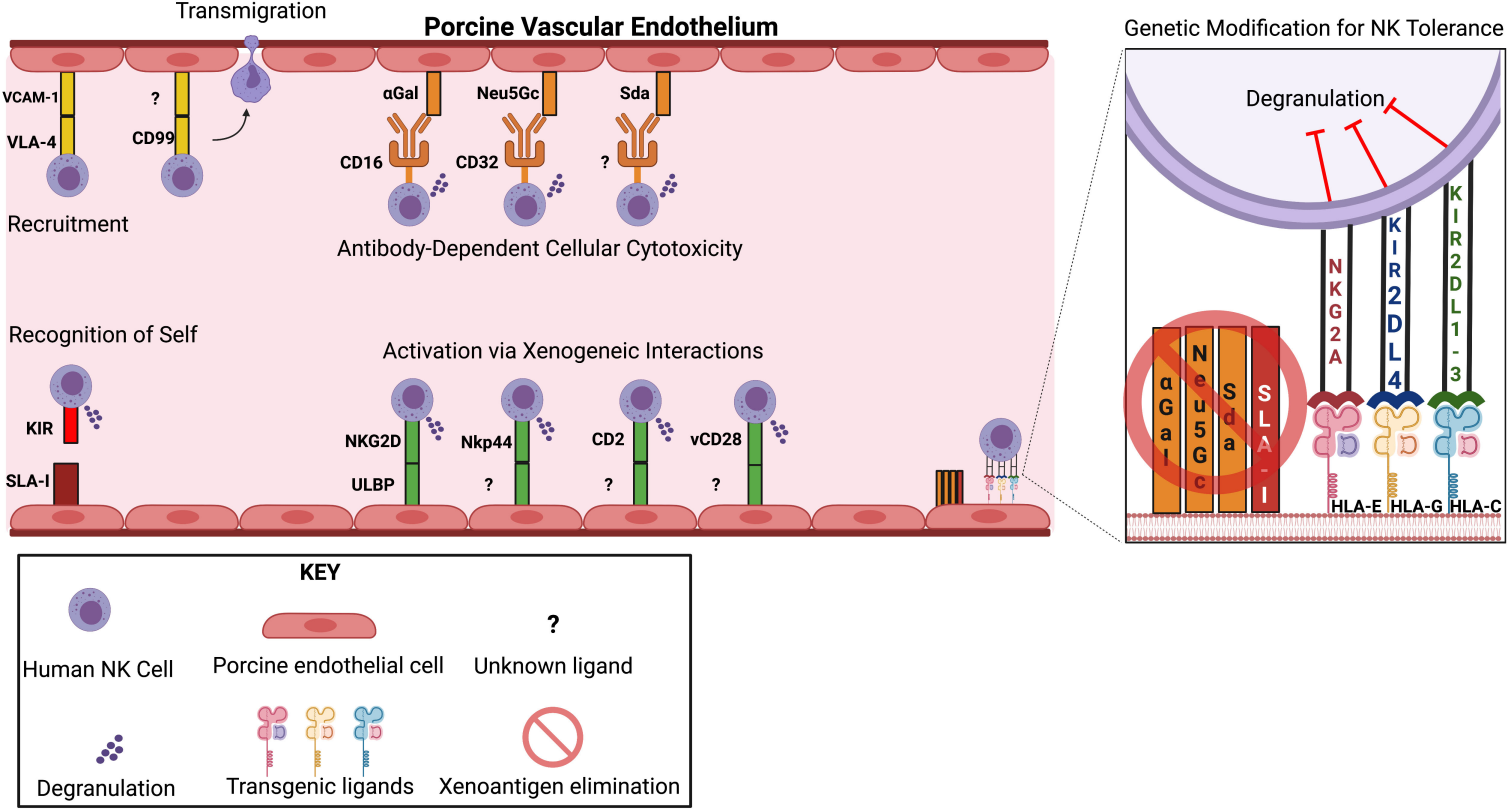
Genetic Modifications that Reduce NK Cell-directed Cytotoxicity. *Top left:* Recruitment occurs due to adhesive interactions between endothelial ligands and NK cell receptors. Transmigration is mediated by interactions between CD99 and unknown ligands on porcine endothelial cells. *Top middle:* Antibody-dependent cellular cytotoxicity (ADCC) present upon NK cell recognition of preformed IgG antibodies directed against the xenoantigens αGal, Neu5Gc, and Sda. *Bottom left:* Failed self-recognition due to non-homology between SLA I and HLA I molecules. *Bottom middle*: NK cell receptor activation results from interactions with unknown porcine ligands. *Right*: Summary of current genetic modification proposed to reduce NK cell-mediated cytotoxicity.

NK cells express various activating and inhibitory receptors that interact with the ligands on target cells (9). The balance between activating and inhibitory signals of NK cells determines NK cell activation or tolerance (14). In classical education (also known as NK licensing), naive hyporesponsive NK cells learn to recognize MHC class I molecules as “self” (15). This knowledge of “self” allows NK cells to activate when target cells are missing MHC ligands. Killer cell immunoglobulin-like receptors (KIR) are a major group of human NK inhibitory receptors for HLA class I molecules. Interaction of NK inhibitory cell receptors KIR2DL4 and CD94 (NKG2A) with non-classical class I molecules HLA-G and HLA-E at the fetomaternal interface results in maternal immune tolerance during pregnancy (16) (**Figure 1**). Activating human NK cell receptors include members of KIR family, NKG2D, natural cytotoxicity receptors such as NKp30, NKP44, NKp46, and the nectin/nectin-like binding receptors DNAM-1 and CRTAM, which are responsible for initiating activating signals (17, 18) (**Figure 1**).

NK cells play a crucial role in influencing immune responses to solid organ allografts. Activated NK cells can kill allogeneic target cells and secrete immunomodulatory chemokines and cytokines, contributing to either rejection or tolerance (19).

In this review, we focus on (i) the dual function of NK cells in rejection and tolerance in allotransplantation, (ii) the state of current research regarding genetic modifications to promote NK cell tolerance in xenotransplantation, and (iii) promising future directions to advance xenotransplantation to the clinical reality.

## NK cells in allotransplantation

Within days of solid organ transplantation, NK cell infiltration has been observed in allografts (20). Historically, acute rejection episodes have been characterized by an increased number of circulating cytotoxic NK cells (21). NK cells are primarily responsible for augmenting the immune response by secreting key pro-inflammatory cytokines, such as TNF-α and INF-γ (22) and recruiting activated lymphocytes (23). Although T cells are the dominant cell type in allograft rejection, fully activated NK cells have been implicated in allograft rejection in the absence of T cells and B cells in mice (24). The early recruitment of immune cells to the graft *via* NK cell cytokine secretion can propagate the acute rejection process, linking the innate and adaptive immune responses (25). NK cell facilitation of rejection is evidenced by simultaneous and significantly elevated expression of NKG2D and its ligands during acute cardiac allograft rejection in mice (26).

Beyond acute rejection, the role of NK cells in chronic rejection has been uncovered in multiple studies. For example, depletion of mouse NK cells by anti-NK1.1 monoclonal antibodies prevented early phase cardiac allograft vasculopathy (CAV) (27). In a RAG1 ^-/-^ murine model (deficient in T and B cells), NK-induced CAV lesions were restored when wild-type helper T (Th) cells were transferred to the recipient, suggesting that NK cells may play a role in chronic rejection in a Th cell-dependent manner (19, 28). A similar role in chronic rejection was noted in a murine lung transplant model as the use of an anti-NK1.1 monoclonal antibody attenuated bronchiolitis obliterans, a form of chronic lung transplant rejection (29). In a retrospective immunogenetic study of human cadaveric kidney allografts, lacking inhibitory combinations of functional recipient KIR and donor HLA ligand led to a higher risk of chronic rejection (30). Collectively these findings support the conclusion that the presence of NK cells promotes chronic allograft rejection. Thus, inhibiting NK cell activation could be beneficial in improving allograft survival.

Additionally, NK cells promote kidney graft rejection independently of immunosuppressant cyclosporine A therapy, which blocks T cell activation through IL-2 inhibition (31). Due to the varying mechanisms of action of immunosuppressants, uniform influence on NK cell cytotoxicity is not seen. Immunosuppressants limit NK cell functions, such as IFN‐γ production, expression of activation/inhibitory and adhesion markers, antibody-dependent cell-mediated cytotoxicity (ADCC), and NK cell proliferation in different manners to different degrees (32). However, identifying potent inhibitors of NK cell functions (methylprednisone and intravenous immunoglobulin) has important implications for their use in promoting xenotransplant acceptance (32).

The role of NK cells in allograft tolerance is more complex, especially given that both recipient and donor NK cells contribute to allograft rejection and tolerance (33–35). Depletion of recipient NK cells has been reported to prolong allograft survival in a murine liver transplantation model (23). Conversely, in a mouse skin allograft model, recipient NK cells were shown to target and destroy donor antigen-presenting cells, ultimately leading to improved skin allograft tolerance (36). A human lung allotransplantation study also showed these opposing actions of rejection and tolerance (37). Given the array of NK cell functions in acute and chronic allograft rejection, a thorough understanding of their precise roles will be essential to designing an immunologically optimized porcine xenograft.

## NK cells in xenotransplantation

Direct contact of human blood with porcine vascular endothelial cells leads to pig endothelial cell injury and secretion of cytokines and chemokines (6, 38, 39). Adhesion molecules, including platelet endothelial cell adhesion molecule, E-selectin (CD62E), P-selectin (CD62P), and vascular cell adhesion molecule-1 (VCAM-1), are upregulated on activated porcine endothelial cells (pEC), and function in recruiting human NK cells (6, 40). Studies showed that interactions of porcine VCAM-1 with human very late antigen-4 (VLA-4) facilitated human NK cell adhesion to pEC (41, 42), and later transendothelial migration was mediated CD99 (43). Manipulating the VCAM-1/integrin adhesion pathway is impractical as the deletion of VCAM-1 was lethal in mice (44).

Preformed human anti-pig antibodies binding to xenoantigens on porcine vascular endothelium initiates the hyperacute xenograft rejection cascade (45, 46). ADCC is a major mechanism for NK cell-mediated cytotoxicity through Fc-receptors (CD16a) binding to IgG1 and IgG3 on pEC and releasing lytic granules (47–49). The αGal-knockout (KO) in pEC line has been shown to significantly suppress ADCC (50, 51). Additional elimination of N-glycolylneuraminic acid (Neu5Gc) (52) and Sda (product of β-1,4 N-acetylgalactosaminyl transferase) (53, 54) carbohydrates and SLA-I xenoantigen in pECs may further inhibit NK cell-mediated ADCC (51). The pivotal work in reducing NK cell hyperacute xenograft rejection is well summarized by Puga Yung et al. (6).

Several studies have been conducted to evaluate the role of NK cells in acute xenograft rejection. In a pig-to-nonhuman primate (NHP) preclinical cardiac xenotransplantation with antibody depletion, Itescu et al. observed greater levels of NK cell and macrophage infiltration in xenografts than in allografts when antibodies were depleted (55), suggesting xenografts experience a more robust cellular immune response even in the absence of antibody-mediated rejection. In a guinea pig-to-rat cardiac xenotransplantation study, continuous depletion of NK cells can prolong xenograft survival (56), further indicating that the absence of NK cells may promote long-term xenograft acceptance. An early and transiently elevated IFN-γ was recognized in pig-to-NHP heart and kidney xenotransplantation when retrospective analyses of NHP sera were performed. Further study indicated that NHP NK cells are the source of early IFN-γ upon the stimulation by both wild-type and αGal-KO pEC *in vitro*, which could amplify inflammation (57). Taken together, these findings suggest that xenografts experience profound cellular rejection, and NK cells play a pivotal role in both executing and amplifying this response.

Given the importance of NK cells in acute xenograft rejection, the interspecies receptor-ligand interaction has been investigated with the goal of manipulating porcine ligands to prevent human NK cell cytotoxicity. Three HLA class I molecules were expressed in an immortalized porcine bone marrow-derived endothelial cell line. HLA-Cw3 provided substantial protection against cytotoxicity of several NK clones, but HLA-A2 and HLA-B27 provided no protection (58). The co-expression of HLA-Cw3 and HLA-Cw4 did not offer additional benefit than when each was expressed alone (59).

The inhibitory role of swine leukocyte antigen-I (SLA-I) towards human NK cells has been investigated in the past with mixed findings. Sullivan et al. reported that SLA-I is unable to efficiently transmit inhibitory signals to human NK cells because amino acid residues critical for the binding to human NK cell inhibitory receptors are altered in SLA-I when compared to HLA class I (60). In contrast, Kwiatkowski et al. found that induction of SLA-I expression on porcine endothelium by TNF-α reduced human NK cell-mediated cytotoxicity (41). Hein et al. reported that decreased SLA-I expression (SLA-I low) did not decrease NK cell activation (61). A recent study demonstrated that SLA-I is not an inhibitory ligand for human NK cells using an immortalized pEC with *SLA-I* gene disruption (5). The absence of inhibitory ligands on pEC triggers human NK cell destruction.

## Future directions

Xenotransplantation differs from allotransplantation in that preventing rejection can be achieved through genetic modification of the donor pig, improving donor-recipient compatibility. A thorough understanding of the NK cell tolerance mechanisms is necessary for developing novel strategies to mitigate NK cell activation in xenotransplantation. To overcome NK cell-mediated acute xenograft rejection, future directions may require emulating the tolerance strategies used at the maternal-fetal interface and the evasion mechanisms employed by viruses (**Table 1**).

**Table 1:** Proposed Directions to Reduce NK Cell-Mediated Rejection.

Model	Target	Expected Outcome
Pregnancy	Co-expression of HLA-E and HLA-G	Addition of inhibitory signal
Viral evasion	UL40 co-expression with HLA-E gpUL18 decoy expression	Promotion of inhibitory signals
Deletion of porcine activating ligands	pULBP1, pCD58, and pCD112 deletion	Deletion of activating ligand
Identification of unknown porcine activating ligands	NKG2D, CD2, NKp44, and DNAM-1 ligands	Identify additional targets for deletion

Future strategies to reduce NK cell-mediated acute xenograft rejection include replicating natural mechanisms of NK cell tolerance in pregnancy and evasion by viruses. Further work is required to identify unknown porcine activating ligands for genetic modifications.

### Introducing inhibitory ligands in pig organs

**Figure 2:**
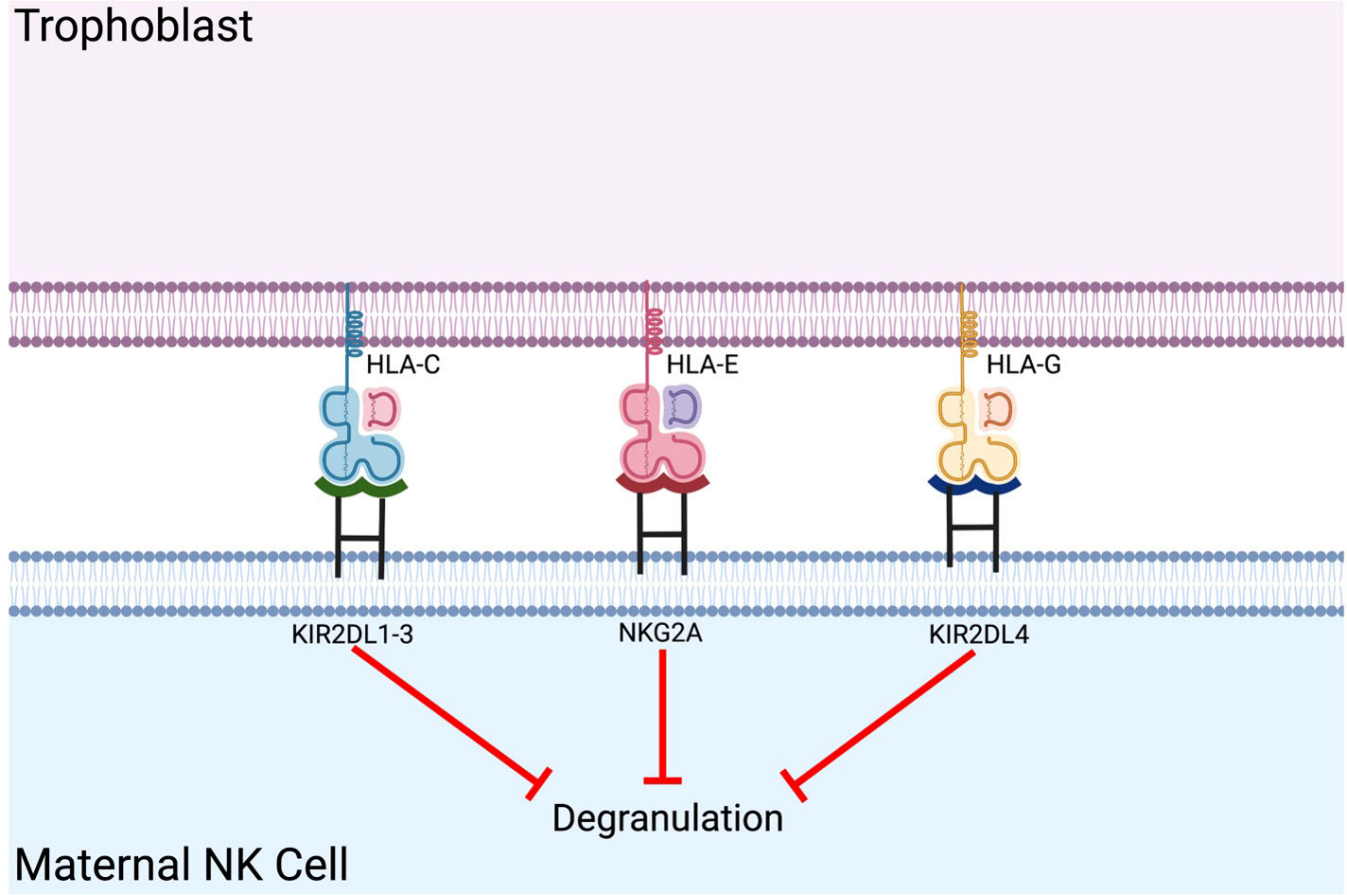
Co-expression of fetal HLA-C, HLA-E, and HLA-G in extravillous trophoblasts provides inhibitory signals to maternal NK cells. Maternal NK cell tolerance is established *via* expression of fetal classical and non-classical HLA class I molecules on the placental extravillous trophoblast.

An example of natural human tolerance is maternal acceptance of the fetus during pregnancy. NK cells constitute the largest proportion of immune cells present in the post-conception endometrium and play a vital role in establishing maternal-fetal immune tolerance. Unique co-expression of fetal HLA-C, HLA-E, and HLA-G in extravillous trophoblasts (EVT) provides inhibitory signals to NK cells (**Figure 2**) (22). Previous studies demonstrated that reduced expression of inhibitory receptors (i.e., KIR2DL1, KIR2DL2, and KIR2DL3) was associated with recurrent spontaneous abortion (62) and that disruption of the NKG2A/HLA-E axis can lead to adverse fetal outcomes in a murine model (63). This specific in-utero environment provides a model for xenotransplantation as the presence of MHC Class I molecules induce an immunotolerant phenotype of maternal NK cells.

Genetically-modified pECs expressing HLA-E demonstrated partial protection from human NK cytotoxicity, and this effect was most pronounced in NK cells with high NKG2A expression (64). This protection was later reproduced in pECs isolated from a double HLA-E β2M transgenic pig (65), confirming the utility of this genetic modification. In an *ex vivo* porcine limb perfusion study with human blood using transgenic pigs co-expressing HLA-E and human CD46 (HLA-E/hCD46), decreased NK cell tissue infiltration was observed (66). Moreover, *in vitro* NK cell-mediated lysis of pECs from HLA-E/hCD46 transgenic pig was significantly reduced compared to pECs from both wild-type and hCD46 alone (66). As a result of transgenic HLA-E expression, *ex vivo* perfusion models have also demonstrated increased survival of porcine lungs (67) and improved function of porcine hearts (68). The ability of HLA-E expression to improve graft compatibility across different organs suggests a generalizable mechanism of immune protection, making it a particularly useful genetic modification for optimizing organ donor pigs.

Expressing HLA-G in porcine aortic endothelial cells inhibits xenogeneic human NK cell-mediated cytotoxicity (69). Dose-dependent soluble HLA-G1 has also been shown to reduce human NK cell-mediated cytotoxicity towards pEC (70). In another study, co-transfection with human β2M enhanced the level of HLA-G1, which led to a significant reduction of NK cell-mediated pEC lysis compared to the HLA-G3 isoform (71)

Given the substantial evidence that both HLA-E and HLA-G expression reduce human NK cell-mediated cytotoxicity, co-expression of HLA-E and HLA-G in xenografts may provide immunoprotection and should be further studied (72). Matsunami et al. discussed the effects of co-transfection of HLA-E and HLA-G into pECs while evaluating NK cell response. Though differences were noted across leader peptides and expression rate, substantial NK cell inhibition was noted in HLA-E and HLA-G co-expression compared to either alone. This synergistic relationship between HLA-E and HLA-G demonstrates the potential use in xenografts (73).

**Figure 3:**
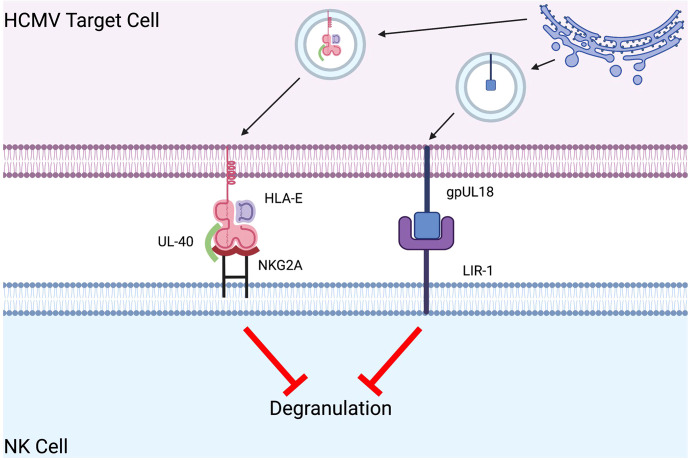
Human CMV evades NK cell destruction by amplifying inhibitory signaling. In human CMV (HCMV) infected target cells, UL40-bound HLA-E bind six-fold greater affinity to the NK cell inhibitory receptor NKG2A than HLA-E alone. HCMV gpUL18 is an MHC I decoy ligand that binds to the inhibitory NK cell receptor LIR-1 with a 1000-fold greater affinity than native MHC I molecules. These interactions result in a significant reduction in NK cell cytotoxicity.

An additional model to reduce NK cell cytotoxicity is noted in viral proteins that increase inhibitory signaling in efforts to circumvent immune destruction. Herpesviridae, a family which includes herpes simplex virus (HSV) and human cytomegalovirus (HCMV), have adapted several unique mechanisms centered around decreasing activating and increasing inhibitory receptor-ligand interactions to evade immune detection by NK cells (74). HCMV evades NK cell destruction by amplifying inhibitory signaling *via* the expression of UL40 and gpUL18 in the endoplasmic reticulum of infected cells (75). UL40-bound HLA-E binds to the NK cell inhibitory receptor NKG2A with greater affinity (6-fold more tightly) than HLA-E alone (**Figure 3**) (76). Similarly, gpUL18, an MHC I decoy ligand, binds to the inhibitory NK cell receptor LIR-1 1000-fold more tightly than native MHC I molecules, resulting in a significant reduction in NK cell cytotoxicity (**Figure 3**) (77–79). The combination of enhanced cell-surface expression of HLA-E and tighter binding to NKG2A results in a marked decrease in NK cell cytotoxicity (80, 81). Repurposing the success of HCMV by expressing these unique viral peptides in xenograft cell lines may induce NK cell tolerance *via* amplification of inhibitory signals (**Table 1**).

### Eliminating activating ligands in pig organs

Another strategy to reduce human NK cell cytotoxicity is eliminating ligands that bind to human NK cell activating receptors. Blocking of activating ligands is a strategy used by HSV-infected cells to downregulate the expression of several ligands of the activating NK cell receptor NKG2D, including MICB (major histocompatibility complex class I polypeptide-related sequence B) and UL-16 binding proteins (ULBP1,2,3) (82, 83). HSV-infected cells transcribe miR-H8, a microRNA that prevents anchoring of NKG2D-activating ligands such as ULBP-2 and ULBP-3 to the cell surface (74, 84–86). HCMV achieves a similar result by expressing the virally encoded UL-16 protein that binds to and sequesters MICB, ULBP1, and ULBP2 (87). Efforts to mimic these mechanisms may provide an approach to promote xenograft acceptance. Nevertheless, findings surrounding these ligand-receptor interactions are still unclear, and further experimentation is necessary.

Lilienfeld et al. confirmed porcine ULBP1 as a ligand for human NKG2D through anti-porcine ULBP1 polyclonal antibody studies (88). Tran et al. subsequently demonstrated additional unknown porcine ligands to NKG2D (89). After generating a ULBP1-KO on a 5GKO pEC line (ULBP1KO/5GKO), our laboratory concluded that porcine ULBP1 is not the dominant ligand for NKG2D as its deletion did not offer a statistically significant decrease in NK cell activation (90). Additional studies are needed to identify functional porcine ligands to human NKG2D in pig cells.

Glycoprotein D of HSV and pseudorabies virus downregulates CD112 expression, a ligand for the activating NK cell receptor DNAM-1, which results in decreased NK cell activation and reduced cytotoxicity (18, 91). Two porcine isoforms of CD112 have been reported (92), but further study is needed to evaluate the role of porcine CD112 in human NK cell activation. Discovery and deletion of porcine activating ligands could promote human NK cell tolerance (**Table 1**). Expressing viral proteins in pigs to reduce activating ligands may represent a complementary approach to inhibiting NK cell-mediated cytotoxicity.

When evaluating an array of activating NK cell receptors (NKp46, 2B4, CD49d, CD48, CD2, and NKG2D), Kim et al. discovered that only CD2 and NKG2D were involved in both cytotoxicity and cytokine-secreting functions against porcine cells (93). It is important to note that complete protection against NK cell-mediated cytotoxicity was only obtained when combinations of activating receptors (NKp44 and NKG2D or CD2 and NKG2D) were blocked (93). Structural protein modeling of human CD2 and porcine CD58 demonstrated a highly conserved interface (94). In fact, blocking with an anti-porcine-CD58 antibody inhibited lysis of porcine cells (95), indicating porcine CD58 is a potential activating ligand for modification. Genetic modifications aimed at elimination of activating ligands will require extensive research to identify the most potent receptor-ligand interactions. Simultaneously maximizing inhibitory signaling will be important to induce NK cell tolerance in xenotransplantation.

## Conclusions

NK cells play a pivotal role in acute xenograft rejection, yet relatively few attempts have been made to overcome this barrier. Inducing human NK cell tolerance by expressing inhibitory ligands and eliminating activating ligands in porcine xenografts *via* genetic engineering approach may provide a novel way to protect xenografts from NK cell-mediated destruction. Understanding the mechanism of cross-species immune recognition and response, developing a novel approach to induce local immune tolerance/acceptance, and guiding the engineering of pigs to meet clinical needs will be our future focus in xenotransplantation.

## Author contributions

BE and PL provided critical guidance in the concept and design of the review paper. KL and AC-N wrote the initial draft of the manuscript. KL and AC-N designed the figures and tables. All authors participated in a critical review of the manuscript. All authors approved the final manuscript.

## Funding

Work on xenotransplantation at Indiana University has been supported by internal funds of the Department of Surgery, in part, with support by the Board of Directors of the Indiana University Health Values Fund for Research Award (VFR-457-Ekser), the Indiana Clinical and Translational Sciences Institute, funded in part by Grant # UL1TR001108 from the National Institutes of Health (NIH), National Center for Advancing Translational Sciences, Clinical and Translational Sciences Award, and NIH NIAID R21AI164002 (PL).

## Conflict of interest

The authors declare that the research was conducted in the absence of any commercial or financial relationships that could be construed as a potential conflict of interest.

## Publisher’s note

All claims expressed in this article are solely those of the authors and do not necessarily represent those of their affiliated organizations, or those of the publisher, the editors and the reviewers. Any product that may be evaluated in this article, or claim that may be made by its manufacturer, is not guaranteed or endorsed by the publisher.

## Abbreviations

αGal, galactose-α(1,3)galactose; AXR, Acute xenograft rejection; β4galNT2, Beta-1,4-N-Acetyl-Galactosaminyltransferase 2; CMAH, Cytidine monophospho-N-acetylneuraminic acid hydroxylase; GGTA1, Alpha-1,3-galactosyltransferase; HLA, Human leukocyte antigen; HXR, Hyperacute xenograft rejection; MHC, Major histocompatibility complex; Neu5Gc, N-glycolylneuraminic acid; NK Cells, Natural Killer Cells; PBMC, Peripheral blood mononuclear cells; pEC, Porcine endothelial cells; SLA, Swine leukocyte antigen; 5GKO, *GGTA1/CMAH/β4galNT2/SLA-Iα/β2-microglobulin KO.*
